# Women in Refugee Camps: Which Coping Resources Help Them to Adapt?

**DOI:** 10.3390/ijerph16203990

**Published:** 2019-10-18

**Authors:** Orna Braun-Lewensohn, Sarah Abu-Kaf, Khaled Al-Said

**Affiliations:** 1 Conflict Management and Resolution Program, Department of Multidisciplinary Studies, Ben-Gurion University of the Negev, Beersheba 8410501, Israel; aks@bgu.ac.il (S.A.-K.); haled70@gmail.com (K.A.-S.); 2 Kay Academic College of Education, Beersheba 84536, Israel

**Keywords:** women, refugees, coping, mental health

## Abstract

The present study aimed to explore the coping resources and mental health of women who have fled Syria to a neighboring European country. To that end, we examined the roles of sociodemographic factors, situational factors, and personal and community sense of coherence (SOC and ComSOC, respectively) in mental-health outcomes. One hundred and eleven refugee women aged 19–70 filled out self-reported questionnaires during August 2018 in a refugee camp in Greece. The questionnaires asked the participants for demographic information (i.e., age, level of education level, and time spent in the camp) and also addressed the situational factors of having received aid from various organizations, appraisal of danger during the war in Syria, and exposure to war experiences, as well as the coping resources of SOC and ComSOC. The results show that time spent in the camp, appraisal of danger, SOC, and ComSOC all play significant roles in predicting the variance of various mental-health outcomes. Together, those factors predict 56% of anxiety, 53% of depression, and 58% of somatization. SOC was also found to mediate the relationships between time spent in the camp and outcome variables, as well as the relationships between the appraisal of danger and the outcome variables. This indicates that SOC is crucial for good adaptation. These results will be discussed in light of the salutogenic theory.

## 1. Introduction

Since 2011, more than 511,000 Syrians have lost their lives to armed conflict and more than 12 million others have been forced from their homes due to the civil war and the penetration of ISIS forces into Syria. To date, more than 5.6 million of those in need have sought refuge outside Syria, mostly in neighboring countries [1]. In fleeing for their lives, refugees face many other stressors related to their war experiences. They seek to resettle in a new country while having to learn a new language. They also face poverty and a lack of resources, which place them at additional risks of violence, discrimination, and social isolation [2].

Based on the salutogenic model [3,4], the present study sought to explore the coping resources of and common mental-health problems (including anxiety, depression, and somatization) among refugee women who have fled Syria to Greece. Specifically, we aimed to examine the roles of educational levels and the time spent in the refugee camp in these women’s adaptation to life in a new country. Based on these sociodemographic factors, we compared the women in terms of several dimensions, namely, a personal sense of coherence (SOC), a community sense of coherence (ComSOC), exposure to the armed conflict, appraisal of danger in the war zone, and whether they had received aid from any of a variety of organizations. In addition, we also wanted to further understand the variables that could explain mental health and adaptation among refugees.

### 1.1. Refugee Women

In times of conflict, women are characterized as powerless victims. During the civil war in Syria, women have faced forms of structural violence from the Syrian regime. Inequalities are emphasized and this affects the ways in which Syrian women experience the process of becoming a refugee. Overall, Syrian women and refugee Syrian women, in particular, are affected by systems of power that marginalize them and their voices [5].

As refugees, these women have been exposed to multiple forms of insecurity and violence. Moreover, a lack of adequate housing adds an additional layer of insecurity and vulnerability to their lives. Some studies have reported that these women face gender-biased violence [6,7]. The lack of suitable housing and access to sanitary facilities also affect the women’s physical and mental health and well-being [6]. Indeed, some studies have indicated that women refugees are an especially vulnerable population, with high rates of depression and other mental-health problems [7].

### 1.2. Experiences of War

Direct exposure to the war in Syria have led the participants in this study to flee their homes and become refugees. Exposure to war events refers to the individuals’ experience of bombs falling and damaging their neighborhoods and surroundings, as well as harm caused to their acquaintances as a result of the war [8]. This type of exposure to violence is likely to increase the risk of psychological problems such as anxiety, depression, and somatization, especially during the first stage of migration [9]. However, results of studies regarding the cumulative exposure to violent political events are inconclusive [10]. While some research on refugees has shown associations between exposure (i.e., the number of events and their intensity) and various psychological problems [11], other studies that have examined war experiences (e.g., one’s community being attacked by rockets/bombs, the experience of someone an individual knows being hurt as result of the war, the experience of having a relative hurt as a result of the war, having been hurt as result of the war, and having had one’s home damaged as result of the war) have indicated that the number of events is not the most significant predictor of post-traumatic stress or other internalizing or externalizing psychological problems [12]. In other work, coping resources such as SOC have been shown to mediate the relationship between exposure to war events and stress-related reactions [13]. Thus, it seems important to evaluate the role of these factors in the context of women refugees, in order to understand their adjustment to life in a new country after having experienced and fled from war.

### 1.3. Appraisal of Danger 

The primary appraisal is the evaluation of the original threat in order to estimate the current threat, and the secondary appraisal is the assessment of the resources one has in order to deal with the stressor [14]. The evaluation of whether a situation represents a threat or a challenge determines the level of arousal and which of the coping resources one has in his/her repertoire that will be drawn upon to deal with the situation [15]. Studies that have examined this variable in the context of war and terror have shown that women are more vulnerable and report more feelings of danger as compared to men [16]. Research has shown that these feelings seem to be an independent predictor of a variety of mental-health symptoms. That is, the stronger the feelings of danger, the higher the threat appraisal and the more intense the mental-health symptoms [8]. 

### 1.4. Receiving Aid

There is a debate as to whether humanitarian aid that focuses mainly on material and social support and is funded by a variety of organizations with political agendas actually benefits the refugees who receive it, or whether it harms or does not affect them. Indeed, most studies in this domain lack empirical evaluations [17]. One study that tried to evaluate whether humanitarian aid mitigates or exacerbates the effects of war on stress reactions was based on two interviews and did not draw a clear conclusion. Additionally, despite the problematic idea of organizations driven by external interests, that work did not suggest relying solely on the refugees’ needs and priorities [18]. A recent study showed that receiving aid from any of a variety of organizations did not play a significant role in reducing psychological problems among refugee youth and had only a moderate relationship with their expectations [12]. The present study tries to add additional knowledge to fill this lacuna in the research by examining the role of aid from a variety of organizations in reducing psychological problems among refugee women.

### 1.5. The Salutogenic Model and Sense of Coherence (SOC)

The secondary appraisal facilitates the exploration of the resources available to the individual to deal with a stressful situation. In this study, we examined the coping resource of SOC, which is rooted in the salutogenic model [3] and is an important concept in positive psychology [19]. The salutogenic model looks for functions of positive qualities rather than healing from sickness [19,20]. Thus, the present study focuses on coping and resilience resources rather than risk factors. The main construct of this model, SOC, is an enduring tendency to see the world as more or less comprehensible, manageable, and meaningful [4]. In accordance with the salutogenic theory, a person with a strong SOC will be more likely to evaluate a particular stimulus as neutral [19]. Therefore, an individual with a strong SOC is less likely than one with a weak SOC to perceive stressful situations as threatening and anxiety-provoking. SOC determines the ability of individuals to use resources that are available to them to promote their well-being [21]. Moreover, SOC includes components that consolidate resilience and enhance subjective mental health [19]. Indeed, numerous studies have shown that SOC may be considered a protective factor that helps to moderate and mediate stress experiences (e.g., [22,23]). Furthermore, Evans and Davis [22] showed that the ways in which SOC acts through family, community, and cultural dimensions can aid successful coping and reduce stress among marginalized and minority ethnic groups. 

### 1.6. Community Sense of Coherence (ComSOC)

From a socio-ecological perspective [24], community is an important resource for various populations and helps them to adjust to new environments [15]. Membership in social groups can act as a social resource [19,22]. In this study, we used the relatively new concept of ComSOC, which has been developed as a culturally sensitive tool. This concept reveals how collective cultures define their SOC through community rather than individual frames, thereby emphasizing societal values. ComSOC embraces the individual’s perception of a community in terms of Antonovsky’s three components: Comprehensibility, manageability, and meaningfulness [25]. Communal resources of comprehensibility, manageability, and meaningfulness enable members of the community to express and to realize themselves, to feel satisfaction, to challenge and have communal interests, and also amplify feelings of affiliation and social connectedness [26,27]. In various studies, ComSOC has been found to be stronger among collectivistic minority cultures than among Western majority cultures and while it has been found to be negatively correlated with psychological problems, it has also helped to explain job satisfaction [28,29]. We assumed that, in the context of Syrian Arab culture, community coherence plays an important role in the refugees’ adjustment to their new environment.

### 1.7. Demographic Factors: Age, Level of Education, and Time Spent in the Refugee Camp

Age serves as a predictor of mental-health problems, with research indicating that older women report more mental-health problems. Moreover, women from traditional and collectivistic societies, such as Arab societies, who are less educated usually report more mental-health symptoms than more educated women [30]. The immigration experience also plays a significant role, with older women and immigrants who are new residents of a country reporting more mental-health problems than younger women who are citizens of that country [31]. 

### 1.8. Research Questions

In accordance with the literature described above, the following research questions and hypotheses were formulated: (1) Are there differences between women who have resided in a refugee camp for up to a year and women who have resided in a refugee camp for between 1 and 2 years, in terms of exposure to war events, feelings/appraisal of danger, having received aid from any of a variety of sources, the coping resources of SOC and ComSOC, and/or the mental-health outcomes of anxiety, depression, and somatization? Based on a recent study, we hypothesized that a longer stay in the camp would be associated with higher levels of psychological problems, more feelings of danger, and weaker SOC. However, based on previous research [12], we did not expect that exposure to war experiences or having received aid would vary with the amount of time spent in the refugee camp. (2) Are there significant differences in the mental-health outcomes of women refugees (i.e., anxiety, depression, and somatization) depending on their educational level and their exposure to war events, feelings/appraisal of danger, receiving aid from any of a variety of sources, and/or the coping resources of SOC and ComSOC? We expected women with higher levels of education to report stronger SOC and fewer mental-health problems [30,32]. In addition, since no information on the independent variables of exposure to war events, feelings/appraisal of danger, or receiving aid was found, we hypothesized that level of education would not be associated with any differences in exposure to war experiences, appraisal of danger, or having received aid. (3) We evaluated a model in which different demographic variables (i.e., age, time spent in the refugee camp, and education level), as well as situational factors of exposure to war events, appraisal of danger, having received aid from organizations (or family or community members), and coping resources were entered as predictors of anxiety, depression, and somatization. We expected age and education level [33], time spent in the camp, exposure to war experiences, appraisal of danger, SOC, and ComSOC [8,12,26] to be significant contributors to the various mental-health outcomes. We hypothesized that while levels of education and the coping resources would have positive effects, age, exposure to war, and a relatively high appraisal of danger would have negative effects. In addition to evaluating the entire model, we also examined the roles of SOC and ComSOC in mediating various relationships between the demographic or situational variables and the outcome variables.

## 2. Materials and Methods

### 2.1. Participants

One hundred and eleven refugee women aged 19–70 (M = 41.01, SD = 11.42), who reported having between 0 and 19 children (M = 3.88, SD = 2.71), participated in this study during August 2018. The women were residing in refugee camps in Greece; 2.7% reported having arrived 1 month prior to the administration of the questionnaire, 22.5% had resided in the refugee camp between 1 and 6 months, 30.6% between 6 and 12 months, and 35.1% reported having resided in the refugee camp for more than a year. Most of the women (77.5%) reported that they had not had a relative in the refugee camp prior to their arrival. Most of these women (74.8%) were Sunnis. In terms of level of education, 3.6% had not had any formal education, 7.3% had only graduated elementary school, 51.8% had only graduated high school, 25.5% had a non-academic higher-education diploma, and 11.8% had an academic degree.

### 2.2. Procedures

Data were collected by self-reported questionnaires during August 2018 in a refugee camp in Greece. Prior to the administration of the questionnaires, the study was evaluated and approved by the university department’s ethics committee (Department of Conflict Management and Resolution, Ben-Gurion University of the Negev). All ethical standards were maintained. All participants were informed that the researchers were interested in their experiences, participation was voluntary, and anonymity was emphasized. The questionnaires were translated into Arabic by an Arabic-language teacher and then reverse-translated into Hebrew to ensure the accuracy of the translation. A researcher who is a native speaker of Arabic approached the women in person, explained to them the nature and aims of the study, and emphasized the voluntary nature of participation and the anonymity of their responses. 

### 2.3. Measures

Demographic characteristics included questions regarding age, number of children, education, ethnicity, when they first entered the refugee camp, and if they had any relatives in the camp prior to their arrival.

*Exposure to war events* was assessed using five yes (1)/no (0) questions that referred to whether the individual’s community had been attacked by rockets/bombs, whether someone the individual knows had been hurt as result of the war, whether a relative had been hurt as a result of the war, whether the individual herself had been hurt as result of the war, and whether the individual’s home had been damaged as result of the war. The answers to the different questions were added up to calculate an index with a potential range of 0–5 (M = 1.35, SD = 0.19).

*Appraisal of danger* was assessed using an index of four questions, each of which was answered using a 5-point Likert scale (1—*not at all*; 5—*very much*). Questions related to how dangerous the situation in Syria was for the study participant, her family, her friends, and civilians in Syria. The mean of the items was calculated to create an index ranging from 1 to 5 (M = 4.36, SD = 0.50).

The variable *receiving aid* was assessed by six questions, each answered using a 5-point Likert scale (1—*not at all*; 5—*very much*). Questions related to receiving aid from family members, Muslim organizations, aid organizations, European governments, and the United Nations. A mean score was calculated to create an index with a range of 1–5 (M = 2.40, SD = 0.54).

*Sense of coherence* (SOC; [4]) was measured using a series of semantic differential items scored on a 7-point Likert-type scale that had anchoring phrases at each end. High scores indicated a strong SOC. An account of the development of the SOC scale and its psychometric properties, showing it to be reliable and reasonably valid, appears in Antonovsky’s writings [4]. In this study, SOC was measured using the short-form scale consisting of 13 items, which was found to be highly correlated to the original long version [4]. The scale includes items such as *“Doing the things you do every day is*” with answers ranging from (1) *“a source of pain and boredom*” to (7) *“a source of deep pleasure and satisfaction.”* In the present study, the Cronbach’s alpha coefficient for the scale was good (α = 0.87).

*Community Sense of Coherence* (ComSOC; [26]). This is a 16-item seven-point Likert-type scale with anchoring phrases at each end. It translates the major themes of Antonovsky’s personal SOC—comprehensibility, manageability, and meaningfulness—into community resources. Items include: *“To what extent do you feel you can influence what’s happening in your community?”*; *“Living in your community gives meaning to your life in a way that other communities couldn’t”*; and “*Do you feel that things that happen in your community have no meaning for you?”*. The Cronbach’s alpha coefficient for this scale in the present study was excellent (α = 0.92).

*Brief Symptom Inventory* [34]. We used the short version of the questionnaire comprised of 18 items, which are rated on a 5-point Likert scale (0—*not at all*; 4—*very much*). The questionnaire examined three areas of psychological and psychiatric problems: somatization, depression, and anxiety. The reliability of the short version of the questionnaire and its three subscales has been reported to be good [35]. Here are examples items from each subscale. Somatization: *“To what extent have you suffered from a feeling of fainting or dizziness?”*. Anxiety: *“To what extent have you suffered from a feeling of stress?”*. Depression: *“To what extent have you suffered from a feeling of depression?*”. In this study, the reliability of the somatization subscale was good (α = 0.87), the reliability of the anxiety subscale was good (α = 0.87), and the reliability of the depression subscale was also good (α = 0.84).

### 2.4. Data Analysis

Statistical analyses were conducted using the statistical software SPSS Version 25, (Routledge, Abingdon, UK). A significance level (*α*) of *p* < 0.05 was chosen. First, the frequencies and percentages of the sample’s demographic characteristics were explored. Then, we ran *t*-tests for independent samples to evaluate the effects of time spent in the refugee camp and levels of education on the different study variables. Finally, a hierarchal regression was performed to investigate the extent to which variance in the dependent variables (i.e., levels of anxiety, depression, and somatization) could be explained by the selected independent variables. We also used the Sobel test [36,37] to evaluate whether SOC and ComSOC mediated the relationships between the different demographic or situational variables and the mental-health outcomes.

## 3. Results

### 3.1. Differences Among Women Who Had Been in the Camp for Different Periods of Time

Our first question related to the comparison of women who had resided up to 1 year in the camp with women who resided in the camp between 1 and 2 years, in terms of our study variables. The results of this analysis are presented in Table 1.

Our analysis revealed some prominent differences, especially in terms of anxiety, depression, and somatization. Contrary to our hypothesis, newcomers reported higher levels of these problems than the veteran residents of the camp. It should be noted that marginal effects were exhibited in personal SOC, with women who had spent more time in the camp reporting stronger SOC. However, it should also be noted that among all of the women, personal SOC and ComSOC were lower than the average of the scale; whereas scores for mental-health outcomes were at the higher ends of those scales.

### 3.2. Differences Among Women According to Their Levels of Education

We then examined differences in the study variables corresponding with the different educational levels of the women. The results of this analysis are presented in Table 2. Contrary to our hypothesis, there were no differences in any of the study variables that corresponded to differences in levels of education. That is, education did not seem to serve as a protective factor in this situation.

### 3.3. Explanation of the Various Mental-Health Outcomes 

Our last question related to the explanation of the mental-health outcomes—anxiety, depression, and somatization—in terms of the different demographic, situational, and coping-resource variables. The results of this analysis are presented in Table 3. It seems that time spent in the camp, appraisal of danger, and the coping resources of SOC and ComSOC are significant in predicting the variance of various mental-health outcomes. Together, those factors predicted 56% of the reported anxiety, 53% of the reported depression, and 58% of the reported somatization. In addition, age was also a significant predictor of somatization. It seems that older women report more somatization. However, overall, it seems that time is a healing factor and that as time passes, the mental health of these women improves. Moreover, the way one perceives a situation and personal and collective resources all play fundamental roles in shaping one’s mental health. 

To evaluate the mediating roles of SOC and ComSOC in the relationships between time spent in the refugee camp or appraisal of danger and the various mental-health outcomes, we ran several Sobel tests. The results indicated that SOC mediated the relationships between time spent in the refugee camp and the appraisal of danger and the outcome variables of anxiety (*z* = 2.00, *p* < 0.05; *z* = 2.79, *p* < 0.01, respectively) and depression (*z* = 2.15, *p* < 0.05; *z* = 2.87, *p* < 0.01, respectively). As for somatization, only the effect of appraisal was mediated by SOC (*z* = 1.99, *p* < 0.05). We also found that ComSOC mediated the role of SOC in the explanation of somatization (*z* = 2.15, *p* < 0.05), underscoring the importance of that variable.

## 4. Discussion

In light of the ongoing civil war in Syria, which has forced millions of Syrians to flee to other countries, this study examined whether and how SOC and ComSOC help Syrian refugee women as they adapt to life in a refugee camp. Rather than examining the topic from a pathogenic point of view, we wanted to understand which coping resources assist these women as they adapt to their new situation. 

Overall, our data indicate that these refugee women are a vulnerable population. Their SOC and ComSOC levels were very low objectively and relative to those of other populations of women around the world who belong to marginalized minority groups [38]. In the same vein, it seems that their mental-health symptoms of anxiety, depression, and somatization are at the higher end of the scales and our findings in this area resemble those of other studies carried out in similar contexts [6,7]. These results are not surprising considering the long civil war from which these women fled. Additionally, these results can be explained by the fact that their new place of residence and their current lives are characterized by insecurity and their futures are uncertain.

Our first research question related to differences between women who had resided in the refugee camp for at least a year (but no more than 2 years) and women who had arrived more recently. Contrary to our hypothesis, our results point to positive adaptation and healing; as time passes, the levels of anxiety, depression, and somatization among these women decrease. Additionally, it seems that the personal resource of SOC becomes stronger over time spent in the camp. This result is in line with those of studies from other places around the world that have shown that when one is torn from one’s home, the first period is a major disturbance, leading to a weakening of various coping-resource systems, but that as time passes, those resources can be recovered [39].

As for the role of education in this setting, contrary to our hypothesis, we found no significant effects of being more or less educated. It seems that in such an extreme context in which women’s lives are in danger, higher education does not provide protection and does not significantly aid women as they adapt to life as refugees.

Our last and most important question related to the role of demographics, situational factors, and personal or communal coping resources in reducing mental-health symptoms, to aid these women’s adaptation to their new environment. In line with our hypothesis, our results show that time spent in the refugee camp and appraisal of danger play significant roles in explaining various mental-health symptoms. In contrast to adolescent Syrian refugees, among whom spending more time in refugee camps has a negative affect [12], for grown women, time spent in the camp has a healing effect. The longer the women had been in the camp, the better mental health they reported. It could be that contrary to adolescent refugees, adult women who have had some time to understand their new environment and deeply comprehend the situation from which they fled can assign new meaning to and better comprehend their potential futures in their new environment despite the difficulties inherent in their situation. Additionally, as previous studies have also indicated (e.g., [8]), our study shows that the way a woman perceives a situation of war and the meaning she assigns to that situation play significant roles in predicting her mental health. Thus, the greater danger she feels, the more negative mental-health symptoms she will report.

Another interesting finding relates to the contribution of age to somatization. In this study, older women reported higher levels of somatization. This finding is in line with our hypothesis and previous studies that have found that the tendency for individuals to present their distress through somatic complaints is common in countries with collectivistic cultures, such as Arab countries [40,41,42]. It seems that the older women were more affected by traditional/collectivistic cultural values and tended to report more somatic symptoms.

Although time spent in the refugee camp and appraisal of danger played significant roles in the explanation of mental-health outcomes, as we hypothesized, it is noteworthy that once the personal resource of SOC was entered into the equation, the importance of the amount of time spent in the refugee camp and appraisal of danger decreased dramatically. These results indicate that SOC and ComSOC have the most important roles in explaining and predicting mental-health outcomes. SOC and ComSOC cancel out or significantly weaken the effects of the above-mentioned variables; stronger SOC leads to better mental health and stronger ComSOC leads to fewer anxiety or somatization symptoms. This study continues a line of studies rooted in positive psychology that have tried to look at factors that promote mental health rather than risk factors that lead to pathogenic outcomes. Thus, it seems that when individuals succeed in finding ways to comprehend and manage their situations, they will enjoy better mental health. Additionally, a community that one can trust and on which one can rely serves as a significant protective factor that promotes adaptation to life in a refugee camp.

A small note regarding the non-significant factors: In this study, the situational variables of exposure to war experiences and having received aid were not found to have any significant effects. These results are congruent with those of a line of studies that have yielded similar results, indicating that variables other than these play significant roles in such situations [8,12].

This study had several limitations that should be acknowledged. First, the data were collected via self-report questionnaires, which may be affected by social-desirability issues [43]. Second, the extent to which women’s experiences of mental-health difficulties converge with external observations, such as clinical reports, remains to be investigated. Third, in the absence of a base rate for the women’s mental-health outcomes prior to the study period, we cannot state with certainty whether or not the observed outcomes are due solely to the impact of exposure to war and the refugee experience. In addition, our research employed a cross-sectional design. All of the variables were measured at the same point in time, so we cannot exclude the possibility that women with higher levels of anxiety, depression, and/or somatization may tend to report low levels of SOC and ComSOC and high levels of appraisal of danger. Future longitudinal studies should shed more light on the nature and the direction of these effects. Finally, a potential degree of sample bias cannot be ruled out as our relatively small sample was not a representative sample of Syrian refugee women. 

## 5. Conclusions

To summarize, the present study examined the roles of SOC and ComSOC in reducing various mental-health outcomes among women who were forced from their homes in Syria. The study participants had resided in a refugee camp in Greece for periods of time ranging from a few weeks to two years. Our study shows that those who had resided in the camps for longer periods of time were better adjusted and exhibited fewer mental-health symptoms and stronger SOC. Moreover, our results also show that SOC and ComSOC play the most important roles in explaining anxiety, depression, and somatization, and also mediate the effects of the amount of time spent in the refugee camp and appraisal of danger on those outcomes.

These results have some practical implications. First, it is very important to strengthen the SOC and ComSOC of refugee women, to enable them to better adapt when confronted with a variety of stressful situations. It is also important that women be integrated into societal processes, in order for them to feel in control of their lives and to strengthen their senses of manageability and comprehensibility. Another way to gain control and increase feelings of manageability is to create routine in the daily life of the inhabitants of the refugee camp. When these women feel that they can influence decisions regarding their lives, they will gain a sense of meaningfulness, which is an important aspect of SOC and which will, in turn, benefit their mental health. 

## Figures and Tables

**Table 1:** Differences among women who had resided in the camp for up to 1 year and women who had resided in the camp for between 1 and 2 years.

	Up to 1 Year *N* = 62		Between 1 and 2 Years *N* = 39		*t*
	*M*	*SD*	*M*	*SD*	
Appraisal of danger (1–5)	4.41	0.42	4.33	0.55	0.77
Having received aid (1–5)	2.42	0.56	2.35	0.55	0.64
Exposure to war events (0–5)	1.34	0.14	1.35	0.27	−0.37
SOC (1–7)	2.56	0.86	2.88	0.75	−1.91 ^^^
ComSOC (1–7)	2.90	1.06	3.17	1.01	−1.25
Anxiety (0–4)	3.43	0.54	3.12	0.58	2.75 **
Depression (0–4)	3.34	0.55	2.95	0.69	3.10 **
Somatization (0–4)	3.27	0.62	2.95	0.78	2.26 *

Note: ^ *p* < 0.06; * *p* < 0.05; ** *p* < 0.01.

**Table 2:** Differences in the study variables among women with different levels of education.

	Up to High School *N* = 69		More Than High School *N* = 41		*t*
	*M*	*SD*	*M*	*SD*	
Appraisal of danger (1–5)	4.32	0.50	4.46	0.48	−1.46
Having received aid (1–5)	2.36	0.45	2.46	0.66	−0.84
Exposure to war events (0–5)	1.35	0.22	1.34	0.14	0.17
SOC (1–7)	2.81	0.77	2.60	0.91	1.26
ComSOC (1–7)	3.01	0.98	3.11	1.11	−0.52
Anxiety (0–4)	3.29	0.52	3.44	0.61	−0.52
Depression (0–4)	3.14	0.57	3.25	0.70	−0.86
Somatization (0–4)	3.18	0.67	3.14	0.70	0.25

**Table 3:** Results of hierarchical multiple regression predicting mental-health outcomes.

		Anxiety					Depression					Somatization			
	*R* ^2^	*B*	*β*	*SE*	*t*	*R* ^2^	*B*	*β*	*SE*	*t*	*R* ^2^	*B*	*β*	*SE*	*t*
**Step 1**	0.09					0.10					0.10				
Age		0.00	0.09	0.01	0.91		0.00	0.05	0.01	0.49		0.01	0.22	0.01	2.21 *
TSC ^1^		−0.36	−0.31	0.12	−3.05 **		−0.42	−0.32	0.13	−3.17 **		−0.39	−0.27	0.14	−2.69 **
Education		−0.02	−0.02	0.12	−0.17		0.02	0.01	0.13	0.14		−0.13	−0.09	0.14	−0.91
**Step 2**	0.22					0.23					0.16				
Age		0.01	0.13	0.00	1.41		0.00	0.08	0.01	0.82		0.01	0.25	0.01	2.60 *
TSC		−0.36	−0.30	0.11	−3.33 **		−0.40	−0.31	0.12	−3.42 **		−0.38	−0.27	0.13	−2.79 **
Education		−0.04	−0.03	0.11	−0.39		0.00	0.00	0.11	−0.02		−0.15	−0.10	0.13	−1.12
Exposure index ^3^		0.01	0.00	0.26	0.02		−0.20	−0.06	0.29	−0.71		−0.10	−0.03	0.33	−0.31
AoD ^2^		0.53	0.44	0.11	4.97 ***		0.60	0.44	0.12	5.08 ***		0.54	0.37	0.13	4.01 ***
Receiving aid		−0.11	−0.11	0.09	1.26		−0.14	−0.13	0.10	−1.42		−0.13	−0.11	0.11	−1.13
**Step 3**	0.25					0.20					0.32				
Age		0.00	0.08	0.00	1.03		0.00	0.04	0.00	0.54		0.01	0.18	0.00	2.45 *
TSC		−0.23	−0.20	0.09	−2.66 **		−0.28	−0.21	0.10	−2.71 **		−0.21	−0.15	0.11	−2.03 *
Education		−0.02	0.02	0.09	−0.25		−0.01	−0.01	0.10	−0.08		−0.11	−0.08	0.10	0.10
Exposure index		0.11	0.04	0.21	0.53		−0.12	−0.04	0.24	0.49		0.05	0.01	0.25	0.20
AoD		0.20	0.16	0.10	1.97 ^^^		0.30	0.22	0.11	2.59 *		0.07	0.05	0.12	0.60
Receiving aid		0.04	0.04	0.08	0.53		0.03	0.03	0.09	0.31		0.07	0.06	0.09	0.78
SOC		−0.18	−0.27	0.07	−2.57 *		−0.28	−0.37	0.08	−3.38 **		−0.18	−0.24	0.09	2.30 *
ComSoc		−0.21	−0.39	0.06	−3.51 **		−0.13	−0.21	0.07	−1.82		−0.33	−0.50	0.07	−4.59 ***

Note: ^ *p* < 0.06; *** *p* < 0.001; ** *p* < 0.01; * *p* < 0.05. 1 Time spent in the camp, 2 Appraisal of danger, ^3^ Exposure to war events.
